# Understanding the relationship between life priorities and life satisfaction in individuals with mental disorders

**DOI:** 10.1002/pcn5.70166

**Published:** 2025-07-30

**Authors:** Daisuke Yoshioka, Takehiko Yamanashi, Kazushi Arima, Naofumi Kajitani, Noriko Kiyama, Minami Sawada, Sizuri Asakura, Akihiko Miura, Ryoichi Matsuo, Koji Komatsu, Hisashi Noma, Masaaki Iwata

**Affiliations:** ^1^ Department of Psychiatry, Faculty of Medicine Tottori University Tottori Japan; ^2^ Department of Psychiatry National Hospital Organization Tottori Medical Center Tottori Japan; ^3^ Department of Psychiatry Matsue City Hospital Shimane Japan; ^4^ Kurayoshi Hospital Tottori Japan; ^5^ Yowa Hospital Tottori Japan; ^6^ Department of Data Science The Institute of Statistical Mathematics Tokyo Japan

**Keywords:** Japan, life satisfaction, mental disorders, text mining, well‐being

## Abstract

**Aim:**

Mental healthcare has recently shifted from focusing solely on treating psychiatric symptoms to enhancing patients’ subjective well‐being. However, research on the factors contributing to life satisfaction among individuals with mental disorders remains limited. This study investigated the life aspects that patients with schizophrenia, depression, and bipolar disorder considered important, their satisfaction with these aspects, and how these factors affect overall life satisfaction.

**Methods:**

A survey was conducted among 152 outpatients at our hospital and six other affiliated hospitals. The participants evaluated their overall life satisfaction, rated the importance of various life aspects, assessed their satisfaction with each, and identified what they considered most important in their lives. A multiple regression analysis revealed the predictors of life satisfaction. Text mining was used to analyze free‐text responses.

**Results:**

The average life satisfaction scores were comparable with those of the general Japanese population. The multiple regression analysis revealed that satisfaction with mental health, romantic relationships, and work were significant predictors of life satisfaction, unlike demographic factors such as age, sex, and diagnosis. The text mining analysis revealed that family was the most frequently mentioned life priority across all diagnostic groups.

**Conclusion:**

By integrating targeted interventions for improving life satisfaction with a deeper understanding of patients’ fundamental values, mental healthcare can provide more comprehensive and practical support.

## INTRODUCTION

Mental disorders are being increasingly recognized as a major cause of disease burden.^1^ Years lived with disability (YLDs) contribute to most of the mental disorder burden, with 125.3 million YLDs (14.6% of global YLDs) being attributable to mental disorders in 2019.^2^ Although it is desirable to elucidate the pathophysiology of mental disorders and develop radical treatments, these goals have yet to be achieved, and many mental disorders follow a chronic course. However, abundant evidence has shown that people with mental illness have the possibility of living a qualified and satisfying life with residual symptoms.^3^, ^4^, ^5^ Some studies have reported that focusing solely on the treatment of mental disorders may alleviate symptoms, but this does not necessarily improve the psychological well‐being of individuals with mental illness.^6^ In recent decades, psychiatric treatment has increasingly focused on improving patients’ subjective well‐being in addition to clinical and functional outcomes.

Life satisfaction is a subjective evaluation of quality of life and a critical component of overall well‐being.^7^ It reflects well‐being across various dimensions, including happiness and spirituality, and is vital in effective disease management.^8^ Psychiatry and mental health professionals are tasked with alleviating patients’ mental health symptoms and enhancing their life satisfaction. To achieve this, it is crucial to assess life satisfaction in individuals with mental disorders and identify the influencing factors. A longitudinal study conducted in New Zealand found that individuals with mental disorders, including major depression, anxiety disorders, suicidality, alcohol dependence, and substance abuse, experienced reduced life satisfaction.^9^ A study conducted in Turkey found no significant difference in life satisfaction between patients with schizophrenia and those with bipolar disorder. Specifically, the study reported mean Satisfaction With Life Scale (SWLS) scores of 16.7 for schizophrenia and 17.4 for bipolar disorder. A negative correlation has been reported between life satisfaction and hopelessness.^10^ Furthermore, a large‐scale population‐based study in Germany found that individuals with major depression and dysthymia had significantly lower SWLS scores than healthy controls, whereas those with bipolar disorder did not significantly differ from the general population.^11^ In addition, a study conducted in Spain involving individuals with severe mental illness—most of whom were diagnosed with schizophrenia or bipolar disorder—reported a mean SWLS score of 21.1, indicating moderate levels of life satisfaction even in clinical populations.^12^

These findings collectively suggest that life satisfaction among individuals with mental disorders is not uniform and may vary not only by diagnostic category but also by geographic and cultural context. However, to our knowledge, no studies in Japan have directly compared life satisfaction across psychiatric diagnoses within a single clinical population. This lack of culturally specific evidence underscores the need for further investigation. In particular, it is essential to identify the factors influencing life satisfaction in clinical settings, considering which aspects of life individuals prioritize and how satisfied they are with those aspects. This study addresses this gap by examining life satisfaction and the life domains prioritized by outpatients with schizophrenia, depression, and bipolar disorder in Japan. Identifying the factors that contribute to life satisfaction is essential for deepening our understanding of patients and establishing a foundation for direct support tailored to their individual needs.

## METHODS

### Participants and procedures

This questionnaire‐based study was conducted between September 2023 and November 2024. The target participants were outpatients from psychiatric departments in the Tottori and Shimane Prefectures. Ultimately, our hospital and six other affiliated hospitals participated in the survey. The inclusion criteria were as follows: (1) aged 15 years or older; and (2) diagnosed with schizophrenia, depression, or bipolar disorder. The exclusion criterion was the inability to respond to the questionnaire, as determined by the attending physician. The participants were informed by their attending doctor about the purpose and method of the study, and their consent was obtained. In addition to the main questionnaire, participants were asked to provide demographic and clinical information, including age, sex, diagnosis, educational level, marital status, living situation, employment status, income, and source of income. The survey was conducted anonymously and on a voluntary basis. Because of this, the total number of patients approached and the response rate could not be determined. All completed responses were included in the analysis, with appropriate methods applied to address missing data. Permission for this study was obtained from the Ethics Committee of Tottori University (approval number: 23A062).

### Assessment tools

#### Satisfaction With Life Scale

The SWLS is a globally used scale for measuring life satisfaction and is easy for participants to respond to. The SWLS was developed by Diener et al.^13^ It consists of five items, and responses are recorded on a seven‐point scale from 1 = *strongly disagree* to 7 = *strongly agree*. The total score ranges from 5 to 35, with higher scores indicating higher life satisfaction. The Japanese version of the SWLS was available as a free‐of charge download on a website provided by Diener.^14^ The internal reliability of the SWLS has been found to be good (around *α* = 0.80) across several studies and samples.^15^

#### Importance and satisfaction in each life aspect among diagnostic groups

Participants rated the importance and satisfaction of 10 life domains using 7‐point Likert scales: Physical Health, Mental Health, Family, Romance, Friends, Money, Work, Social Status, Learning, and Hobbies. Importance was rated from 1 = *not at all important* to 7 = *very important*, and satisfaction from 1 = *very dissatisfied* to 7 = *very satisfied*, reflecting how important each aspect was in their life and how satisfied they were with it at present. In addition, participants were asked to freely describe the most important aspect of their life and rate their satisfaction with it using the same scale. These items were developed specifically for this study.

#### Statistical analysis

To analyze the patient background and the importance and satisfaction of life aspects, one‐way analysis of variance (ANOVA) was applied for normally distributed variables, and the Kruskal–Wallis test was used for non‐normally distributed variables. Chi‐square tests were performed for categorical variables. Post‐hoc comparisons for Kruskal–Wallis were conducted using Mann–Whitney *U*‐tests, and pairwise Chi‐square tests were applied for categorical data. Holm correction was used for multiple comparisons. In these analyses, cases with missing diagnostic information were excluded, and other missing values were handled using pairwise deletion. To analyze the factors associated with life satisfaction (SWLS scores), we performed a multiple linear regression analysis, modeling SWLS scores as the outcome variable, and diagnosis, age, sex, and satisfaction scores for various life aspects (e.g., Physical Health, Mental Health, Work, and Social Status) as explanatory variables. For this regression analysis, all available responses were included, and missing values—including diagnosis—were handled using multiple imputations by chained equations with 200 imputations.^16^ To assess multicollinearity among the explanatory variables in the multiple linear regression model, we calculated the variance inflation factor (VIF).

#### Text mining analysis

First, missing values were removed, and responses were tokenized using a Japanese morphological analysis tool. Next, the original Japanese responses were automatically categorized based on their semantic content using a data‐driven procedure. If a response matched multiple categories, it was classified based on primary semantic content, whereas ambiguous responses were labeled “Other.” No human judgment or rater‐based coding was applied to ensure objectivity and consistency. Finally, category frequencies were calculated and visualized using a pie chart. After classification, the Japanese free‐text responses were translated into English using a large‐language model (ChatGPT, OpenAI), as shown in Table S1. All responses had been fully anonymized prior to translation, and no personally identifiable information was included in the content processed by the model. To avoid introducing subjective interpretation, no human editing or verification was performed on the translated responses. All text mining processes, including tokenization, classification, and visualization, were performed using Python (Version 3.11.8).

## RESULTS

### Characteristics of the study participants

We received responses from 78 patients with schizophrenia, 61 patients with depression, and 10 patients with bipolar disorder. Three respondents did not report their diagnosis and were, therefore, excluded from the sociodemographic analysis. Their sociodemographic characteristics are shown in Table 1. There were no significant differences in age, sex, living situation, education level, employment status, or source of income among the three diagnostic groups. However, marital status differed significantly, with individuals with schizophrenia more often being never married. The mean SWLS score for each diagnostic group was approximately 18, with no statistically significant differences. However, given the small number of participants in the bipolar disorder group (*n* = 10), comparisons across diagnostic groups should be interpreted with caution.

**Table 1:** Sociodemographic characteristics of study participants.

	Schizophrenia	Depression	Bipolar disorder	*p‐*values	Missing data
	*n* = 78	*n* = 61	*n* = 10		*n* (%)
**Age, years (mean ± SD)**	53.6 ± 12.7	58.1 ± 17.3	57.8 ± 17.9	0.19	1 (0.67%)
**Sex**					0 (0.0%)
Male	38 (48.7%)	27 (44.2%)	4 (40.0%)		
Female	40 (51.3%)	34 (55.7%)	6 (60.0%)	0.8	
**Marital status**					1 (0.67%)
Never married	48 (62.3%)**	18 (29.5%)**	3 (30.0%)		
Married or in a relationship	21 (27.3%)**	35 (57.4%)**	4 (40.0%)		
Separated, divorced or widowed	8 (10.4%)	8 (13.1%)	3 (30.0%)	8.70e‐04	
**Living situation**					0 (0.0%)
Living alone	24 (30.0%)	11 (19.4%)	4 (40.0%)		
Living with family or partner	51 (66.2%)	50 (80.6%)	6 (60.0%)		
Living in an institution	3 (3.8%)	0 (0.0%)	0 (0.0%)	0.13	
**Education**					3 (2.0%)
Junior high school graduate	14 (18.4%)	4 (6.7%)	1 (10.0%)		
Senior high school graduate	38 (50.0%)	31 (51.7%)	7 (70.0%)		
University graduate or vocational school diploma	24 (31.9%)	25 (41.7%)	2 (20.0%)	0.2	
**Employment status**					1 (0.67%)
Employed	28 (36.4%)	22 (36.1%)	2 (20.0%)	0.58	
Unemployed	49 (63.6%)	39 (63.9%)	8 (80.0%)		
**Source of income**					1 (0.67%)
Employment income (salary)	22 (28.8%)	20 (32.8%)	3 (30.0%)		
Non‐employment income^a^	55 (71.4%)	41 (67.2%)	7 (70.0%)	0.87	
**Monthly income** b **(Median [IQR])**	70,000 [4500–100,000]*	100,000 [60,000–200,000]*	80,000 [60,000–140,000]	0.04	31 (20.8%)
**Satisfaction With Life Scale (mean ± SD)**	18.0 ± 7.0	18.2 ± 6.5	18.1 ± 7.4	0.98	13 (8.7%)

^a^ Non‐employment income includes pensions, public assistance, and financial support from family members or others.

^b^ Income is shown in Japanese yen (¥). Interquartile range (IQR) is presented as 1st quartile–3rd quartile.

* *p* < 0.05

** *p* < 0.01.

### Importance in each life aspect among diagnostic groups

**Figure 1:**
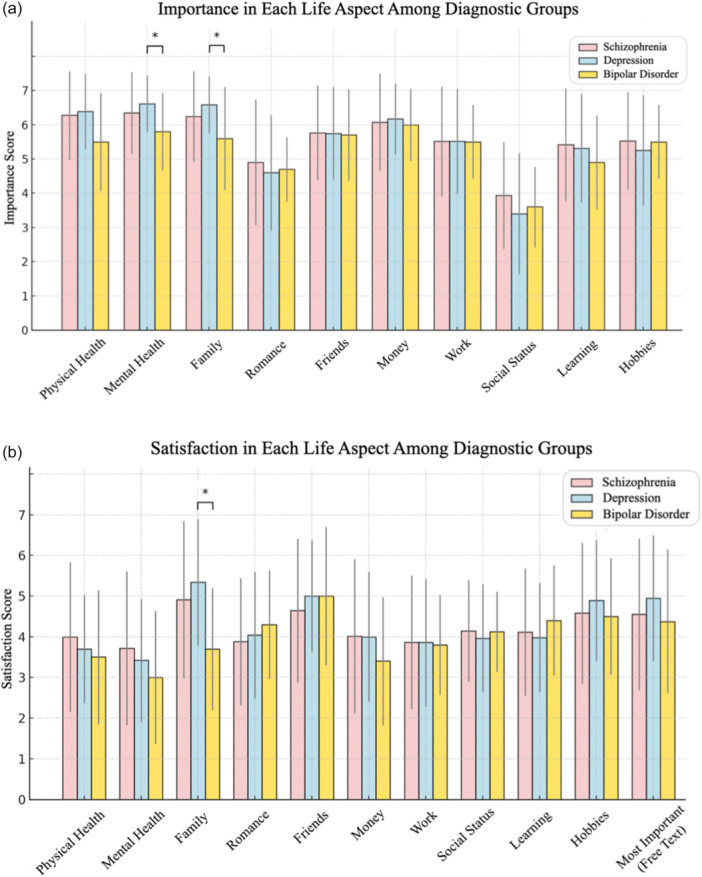
Importance and satisfaction in each life aspect among diagnostic groups. Bar graphs showing (a) importance and (b) satisfaction ratings for 10 life domains among individuals diagnosed with schizophrenia, depression, or bipolar disorder. Each domain was rated on a 7‐point Likert scale, where higher scores reflect greater importance or higher satisfaction. In panel (b), the item labeled “Most Important” represents satisfaction ratings for participants’ personally identified top life priority, as reported in a free‐text response. Bars represent group means, and error bars indicate standard deviations. As all variables were non‐normally distributed, group comparisons were conducted using the Kruskal–Wallis test followed by post‐hoc Mann–Whitney *U*‐tests; Holm correction was applied for multiple comparisons. Statistical significance is indicated by * (*p* < 0.05). Family and Mental Health showed significant group differences in importance ratings. Money was consistently rated as highly important across all groups, whereas Social Status was rated as least important. In terms of satisfaction, Family satisfaction was significantly lower in the bipolar disorder group. Satisfaction scores for Physical Health, Mental Health, and Work tended to be lower across all groups.

As shown in Figure 1a, the importance scores for various life aspects were similar across the three diagnostic groups, except for Mental Health and Family, which showed significant differences between the depression and bipolar groups. While the most important aspect varied by group, Money was consistently rated as highly important across all groups, with mean scores exceeding 6. Conversely, the average score for Social Status was below the median value of 4, suggesting that social status was perceived as a less important aspect of life.

### Satisfaction in each life aspect among diagnostic groups

Figure 1b shows the satisfaction scores for various aspects of life in the diagnostic groups. Overall, there were no major differences in the satisfaction scores among the groups. However, in the Family domain, patients with bipolar disorder reported significantly lower satisfaction than those with depression. Moreover, among all groups, Physical Health, Mental Health, and Work were the only aspects with mean satisfaction scores at or below the median value of 4, indicating lower satisfaction.

#### Associations between satisfaction in life aspects and overall life satisfaction

The results of the multiple regression analysis, adjusted for age, sex, and diagnostic group, focusing on life satisfaction, are presented in Table 2. All predictors in the regression model had VIF values below 2.5, confirming that multicollinearity was not a concern. The full list of VIF values is presented in Table S2. Significant positive associations with the SWLS were observed for Mental Health, Romance, and Work. Additionally, age, sex, and diagnosis were not significantly associated with the SWLS. These findings suggest that psychological and occupational factors, rather than demographic characteristics or diagnostic categories, play critical roles in determining life satisfaction.

**Table 2:** Results of the regression model for Satisfaction With Life Scale.

	Estimate	SE	*t*	*p‐*values	95% CI
**Intercept**	0.28	2.50	0.11	0.91	(−4.67, 5.23)
**Age**	0.02	0.03	0.61	0.54	(−0.04, 0.07)
**Sex (vs. male)**	0.53	0.86	0.61	0.54	(−1.18, 2.24)
**Diagnostic groups**					
Depression (vs. schizophrenia)	0.16	0.91	0.17	0.86	(−1.64, 1.95)
Bipolar disorder (vs. schizophrenia)	1.62	1.74	0.93	0.35	(−1.82, 5.07)
**Satisfaction in life aspects**					
Physical Health	−0.67	0.37	−1.81	0.07	(−1.39, 0.06)
Mental Health	1.92	0.35	5.49	2.29e‐07	(1.23, 2.61)
Family	0.40	0.31	1.28	0.21	(−0.22, 1.02)
Romance	1.05	0.35	2.99	3.40e‐03	(0.36, 1.75)
Friends	−0.08	0.32	−0.24	0.81	(−0.72, 0.56)
Money	0.26	0.37	0.70	0.48	(−0.47, 0.99)
Work	0.99	0.35	2.81	5.90e‐03	(0.29, 1.68)
Social Status	0.50	0.41	1.21	0.23	(−0.32, 1.31)
Learning	−0.54	0.39	−1.38	0.17	(−1.32, 0.23)
Hobbies	0.14	0.32	0.42	0.67	(−0.50, 0.78)
Free (Most important)	0.26	0.29	0.89	0.38	(−0.31, 0.83)

Abbreviations: 95% CI, 95% confidence interval; Estimate, Estimated beta coefficient; SE, Standard error of the estimate; *t*, *t* statistic.

#### Categorization of life priorities through text mining

**Figure 2:**
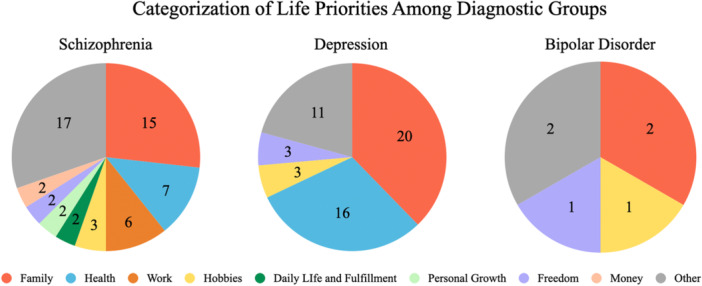
Categorization of life priorities among diagnostic groups. Pie charts showing the categorization of life priorities reported in free‐text responses by participants with schizophrenia, depression, and bipolar disorder. Participants answered the question, “What do you consider most important in life?” Responses were analyzed using text mining through automatic semantic classification. Categories were generated through a data‐driven procedure without human coding. Family was the most frequently mentioned priority across all groups, while Health ranked second in the schizophrenia and depression groups. A large proportion of responses were categorized as Other, indicating diverse individual values. Numbers in each segment indicate the number of participants per category.

Responses to the question “What do you consider most important in life?” were analyzed using text mining techniques, and the results for each diagnostic group are presented in pie charts (Figure 2). The translated patient free‐text responses and their classifications are available as supplemental data (Table S1). The analysis revealed that family was the most prioritized category across all diagnostic groups, including those with schizophrenia, depression, and bipolar disorder. This finding suggests that the family is a crucial source of support for individuals with mental disorders. Additionally, Health was a particularly emphasized category in both the schizophrenia and depression groups, ranking as the second most common category in both groups. Furthermore, the proportion of responses categorized as Other was relatively high, indicating that a substantial portion of free‐text responses did not clearly align with any category. This finding highlights the diversity and individuality of the values participants prioritize.

## DISCUSSION

To the best of our knowledge, this is the first study to investigate life satisfaction in patients with schizophrenia, depression, and bipolar disorder within a single study.

Interestingly, despite the common perception that mental disorders negatively affect life satisfaction, the average SWLS score in our sample was comparable to a previously reported normative average of healthy Japanese individuals (18.9).^17^ Although healthy controls were not included in our study—a notable limitation—the similarity between our participants’ average score and this normative value is particularly noteworthy. Two complementary frameworks may help explain this finding. Adaptation theory describes how individuals maintain well‐being through emotional regulation, coping strategies, and cognitive flexibility in response to adversity.^18^ In parallel, response shift refers to changes in internal standards or values that occur during such psychological adjustment, leading individuals to redefine what life satisfaction means to them.^19^, ^20^ Importantly, response shift is not merely a measurement artifact but may itself be a target for interventions aimed at enhancing quality of life.^21^ Rather than reflecting a lack of distress, high SWLS scores among individuals with mental disorders may indicate successful psychological adaptation and reappraisal.

The analysis of the importance of life aspects showed that the general trend was similar across the diagnostic groups, indicating that patients with schizophrenia, depression, and bipolar disorder share similar priorities in life. However, Mental Health and Family were rated significantly higher in the depression group than in the bipolar disorder group. This suggests that individuals with depression may place greater emphasis on mental well‐being and family relationships than those with bipolar disorder. Across all diagnostic groups, the importance score for Money remained consistently high, indicating that financial stability is universally valued regardless of specific mental disorders. Mental health problems, financial difficulties, and social isolation frequently co‐occur and reinforce each other through a self‐perpetuating cycle. Previous studies have reported that financial support for individuals with severe mental illness can lead to improvements in psychiatric symptoms.^22^ In conjunction with our findings, this underscores the need to consider financial stability a critical component of psychiatric care for individuals with mental disorders. By contrast, Social Status was consistently rated as less important across all groups. This may reflect Japanese cultural values, emphasizing collectivism and harmony over individualism and social status. However, this may also reflect the challenges faced by individuals with mental disorders in engaging with environments that emphasize social status, as they may have limited opportunities to interact with such social structures.

Satisfaction with various aspects of life was also similar across diagnostic groups, suggesting that mental illness may not substantially alter perceptions of satisfaction in these areas. Overall, the satisfaction scores were moderate, indicating that individuals with mental health disorders often experience only partial fulfillment in many aspects of their lives. However, satisfaction with Family was significantly lower in the bipolar disorder group than in the depression group. This suggests that individuals with bipolar disorder face unique challenges in family relationships, possibly due to factors such as interpersonal difficulties, mood instability, or the impact of their condition on family dynamics. Notably, satisfaction with Physical Health, Mental Health, and Work was consistently at or below the midpoint score of 4, highlighting the critical areas of concern. The management of physical health in individuals with mental disorders has long been recognized as a critical issue,^23^ and this survey highlights the significant challenges in this area. Low satisfaction with work may also reflect broader social challenges, such as employment difficulties and workplace stigma faced by individuals with mental health disorders. Low satisfaction with mental health among individuals with mental disorders may reflect the direct impact of their condition, including persistent symptoms, as well as the effects of societal stigma, which lower their self‐esteem and well‐being. Additionally, physical health issues and social factors, such as challenges in relationships and work, further contribute to this dissatisfaction.

Multiple regression analysis revealed that satisfaction with Mental Health, Romance, and Work were significant predictors of overall life satisfaction. One study suggested that interest in romance and sexual behavior among those with schizophrenia and other mental disorders was largely similar to that of the general population.^24^ However, another report indicated that the marriage rate among individuals with schizophrenia and other mental disorders was as low as 8%, highlighting a substantial gap between interest in romantic relationships and actual relationship status.^25^ Romantic relationships, marriage, and parenting are areas of strong interest and hope for patients and their families and often contribute to the recovery process.^24^ Based on the findings of this study, psychiatric care should focus on romantic relationships of individuals with mental disorders, recognizing their importance in both recovery and overall well‐being.

The importance of employment for individuals with mental disorders has been widely recognized.^26^ Employment provides financial independence and greater control over life, making it a crucial factor in improving life satisfaction. It also deepens social connections, prevents isolation, and offers a sense of purpose and hope for the future.

A key feature shared by romantic relationships and employment is their role in fostering connections with others. Establishing social connections and cultivating a sense of belonging are essential for good health, particularly mental health.^27^, ^28^ Regarding psychological functioning, previous research suggests that fostering a sense of belonging is more important than merely increasing social support.^29^ In psychiatric interventions, shifting towards approaches prioritizing the cultivation of a sense of belonging may lead to meaningful improvements in patient life satisfaction. Furthermore, mental health, significantly associated with life satisfaction in this study, is a multifaceted concept that encompasses symptom management, social connections, a sense of belonging, and role fulfillment. While improving mental health is inherently complex, fostering a sense of belonging and strengthening social connections may serve as practical and effective strategies for enhancing mental health and the overall quality of life. Interestingly, factors such as age, sex, and diagnostic group were not significant predictors of life satisfaction. This suggests that personal experiences and satisfaction with specific life aspects are more crucial in shaping life satisfaction than demographic or clinical characteristics. These findings emphasize the need for a holistic approach that respects individual values and promotes social connections to enhance long‐term well‐being.

The text mining analysis of responses to “What do you consider most important in life?” revealed Family as the most prioritized category across all diagnostic groups; Health was also highly valued in the schizophrenia and depression groups. In contrast, multiple regression analysis with SWLS as the dependent variable showed significant associations with Mental Health, Romantic Relationships, and Work, whereas Family was not directly associated. This apparent discrepancy may reflect the conceptual distinction between core values and momentary evaluations of life satisfaction. Core values, such as Family, tend to remain stable over time and change only in response to major life events, whereas life satisfaction is more volatile and responsive to current emotional and situational factors. As such, domains like Romantic Relationships, Work, and Mental Health, which provide emotional and practical rewards in everyday life, may have a stronger impact on how individuals evaluate their life satisfaction at a given moment—even if Family remains a central guiding value.

Moreover, a significant number of responses were categorized as Other, indicating that patients’ perspectives on what is most important in life are highly diverse. These responses included elements such as art, religion, and social contributions, reflecting that values are diverse and shaped by individual life experiences and cultural backgrounds.

This study has several limitations. First was the relatively small number of participants with bipolar disorder compared to those with schizophrenia and depression. This disproportionate group size not only limits the generalizability of the findings related to bipolar disorder but also affects the reliability of direct comparisons across diagnostic groups. Therefore, any interpretation of group differences should be made with caution. Future studies should include a more balanced representation of different diagnostic groups to ensure a more comprehensive understanding of life satisfaction and its associated factors across mental health conditions. Second, while we used ChatGPT to translate the free‐text responses into English to minimize human bias, we acknowledge that this approach may lead to inconsistencies or inaccuracies in nuanced expressions. This limitation should be considered when interpreting the translated data.

Overall, this study investigated factors associated with life satisfaction among individuals with mental disorders. Interestingly, our findings suggest that having a mental disorder does not necessarily lead to significantly lower life satisfaction, as the scores in our sample were comparable to those of healthy individuals. Furthermore, satisfaction with mental health, romantic relationships, and work plays a particularly important role in determining life satisfaction. The study also revealed that personal values are diverse, with family and health emerging as primary priorities. These findings indicate that psychiatric care should incorporate interventions that address factors influencing life satisfaction and a commitment to respect patients’ fundamental values. By adopting this comprehensive and patient‐centered approach, mental healthcare can contribute to improving the overall quality of life of individuals with mental disorders.

## AUTHOR CONTRIBUTIONS

Daisuke Yoshioka and Takehiko Yamanashi designed the study and drafted the manuscript. Daisuke Yoshioka and Hisashi Noma contributed to data analysis. Daisuke Yoshioka, Takehiko Yamanashi, Kazushi Arima, Naofumi Kajitani, Noriko Kiyama, Minami Sawada, Sizuri Asakura, Akihiko Miura, Ryoichi Matsuo, Koji Komatsu, and Masaaki Iwata contributed to patient recruitment. Masaaki Iwata critically revised the manuscript. All coauthors approved the final version of the manuscript for publication.

## CONFLICT OF INTEREST STATEMENT

The authors declare no conflicts of interest.

## ETHICS STATEMENT

Permission for this study was obtained from the Ethics Committee of Tottori University (approval number: 23A062). All participants provided written informed consent prior to participation. The study was conducted in accordance with the Declaration of Helsinki.

## PATIENT CONSENT STATEMENT

Informed consent was obtained from all participants prior to their inclusion in the study.

## CLINICAL TRIAL REGISTRATION

This study is an observational survey and was not registered as a clinical trial.

## Supporting information

Supplementary table 1.

Supplementary Table 2 (VIF).

## Data Availability

The datasets generated and analyzed during the current study are not publicly available due to patient privacy concerns but are available from the corresponding author on reasonable request.
